# Supplementary material for: Understanding cauda equina syndrome: protocol for a UK multicentre prospective observational cohort study
Source: BMJ Open. 2018 Dec 14;8(12):e025230. doi: 10.1136/bmjopen-2018-025230 (PMC6303568; doi:10.1136/bmjopen-2018-025230)
Supplement: Author's manuscript [file bmjopen-2018-025230.draft_revisions.pdf]

BMJ Open is committed to open peer review. As part of this commitment we make the peer review history of every article we publish publicly available.

When an article is published we post the peer reviewers' comments and the authors' responses online. We also post the versions of the paper that were used during peer review. These are the versions that the peer review comments apply to.

The versions of the paper that follow are the versions that were submitted during the peer review process. They are not the versions of record or the final published versions. They should not be cited or distributed as the published version of this manuscript.

BMJ Open is an open access journal and the full, final, typeset and author-corrected version of record of the manuscript is available on our site with no access controls, subscription charges or pay-per-view fees (http://bmjopen.bmj.com).

If you have any questions on BMJ Open's open peer review process please email editorial.bmjopen@bmj.com

BMJ Open

Understanding Cauda Equina Syndrome: protocol for a multi-centre prospective observational cohort study

Journal:	*BMJ Open*
Manuscript ID	bmjopen-2018-025230
Article Type:	Protocol
Date Submitted by the Author:	05-Jul-2018

Complete List of Authors:	Woodfield, Julie; Western General Hospital, Department for Clinical Neurosciences; The University of Edinburgh, Centre for Clinical Brain Sciences<br>Horitzauer, Ingrid; Western General Hospital, Department for Clinical Neurosciences; The University of Edinburgh, Centre for Clinical Brain Sciences<br>Jamjoom, Aimun; The University of Edinburgh, Centre for Clinical Brain Sciences; Aberdeen Royal Infirmary, Department of Neurosurgery<br>Pronin, Savva; The University of Edinburgh, Centre for Clinical Brain Sciences<br>Srikandarajah, Nisaharan; Walton Centre NHS Foundation Trust, Department of Neurosurgery<br>Poon, Michael; Western General Hospital, Department for Clinical Neurosciences<br>Roy, Holly; Derriford Hospital, South West Neurosurgery Centre<br>Demetriades, Andreas; Western General Hospital, Department for Clinical Neurosciences<br>Sell, Philip; Queens Medical Centre, Centre for Spinal Studies and Surgery<br>Eames, Niall; Royal Victoria Hospital, Trauma and Orthopaedics<br>Statham, Patrick; Western General Hospital, Department for Clinical Neurosciences<br>Research Collaborative, British Neurosurgical Trainee ; British Neurosurgical Trainee Research Collaborative, British Neurosurgical Trainee Research Collaborative

Keywords:	cauda equina syndrome, spinal surgery, cohort study, outcomes

SCHOLARONE™
Manuscripts

TITLE

Understanding Cauda Equina Syndrome: protocol for a multi-centre prospective observational cohort study

AUTHORS

Julie Woodfield[1,2], Ingrid Hoeritzauer[1,2], Aimun AB Jamjoom[2,3], Savva Pronin[2], Nisaharan Srikandarajah[4], Michael Poon[1], Holly Roy[5], Andreas K Demetriades[1], Phil Sell[6], Niall Eames[7], Patrick Statham[1], British Neurosurgical Trainee Research Collaborative (BNTRC)

1. Department of Clinical Neurosciences, Western General Hospital, Edinburgh, UK
2. Centre for Clinical Brain Sciences, University of Edinburgh, Edinburgh, UK
3. Department of Neurosurgery, Aberdeen Royal Infirmary, Aberdeen, UK
4. Neurosurgery, The Walton Centre NHS Foundation Trust, Liverpool, UK
5. South West Neurosurgical Centre, Derriford Hospital, Plymouth, UK
6. Centre for Spinal Studies and Surgery, Queens Medical Centre, Nottingham, UK
7. Department of Spinal Surgery, Royal Victoria Hospital, Belfast, UK

Corresponding Author:

Julie Woodfield, Neurosurgery Specialty Trainee, Department of Clinical Neurosciences, Western General Hospital, Crewe Road South, Edinburgh. EH4 2XU

julie.woodfield@nhs.net

0131 537 1000

WORD COUNT

3245

For peer review only - http://bmjopen.bmj.com/site/about/guidelines.xhtml
For peer review only

ABSTRACT

Introduction

Cauda equina syndrome (CES) is a potentially devastating condition caused by compression of the cauda equina nerve roots. This can result in bowel, bladder and sexual dysfunction plus lower limb weakness, numbness, and pain. CES occurs infrequently but has serious potential morbidity and medico-legal consequences. This study aims to identify and describe the presentation and management of patients with CES in the United Kingdom (UK).

Methods and Analysis

Understanding Cauda Equina Syndrome (UCES) is a prospective, collaborative, multicentre cohort study of adult patients with confirmed CES managed at specialist spinal centres in the UK. Participants will be identified using neurosurgical and orthopaedic trainee networks to screen referrals to spinal centres. Details of presentation, investigations, management and service usage will be recorded. Both patient and clinician reported outcome measures will be assessed for one year after surgery. This will establish the incidence of CES, current investigation and management practices, and adherence to national standards of care. Outcomes will be stratified by clinical presentation and patient management. Accurate, up to date information about the presentation, management, and outcome of patients with cauda equina syndrome will inform standards of service design and delivery for this important but infrequent condition.

Ethics and Dissemination

UCES received a favourable ethical opinion from the South East Scotland Research Ethics Committee 02 (Reference: 18/SS/0047; IRAS ID: 233515). All spinal centres managing patients with CES in the UK will be encouraged to participate in UCES. Study results will be published in medical journals and shared with local participating sites.

Registration Details

UCES is sponsored by NHS Lothian (Reference: AC18017). UCES is registered at ClinicalTrials.gov (Record 160318) and pending registration with ISRCTN.

For peer review only - http://bmjopen.bmj.com/site/about/guidelines.xhtml

KEYWORDS

Cauda Equina Syndrome, cohort study, outcomes, spinal surgery

ARTICLE SUMMARY

Strengths and Limitations of this Study

- This UK wide study will be the largest prospectively established cohort of patients with CES.
- The collection of detailed clinical data will describe the range of presentations treated as CES in the UK in current practice and allow stratification of findings by clinical presentation.
- Validated outcome measures will be used to assess pain, disability, and bladder, bowel, and sexual function one year after treatment.
- Participant identification and recruitment will be efficiently carried out using trainee research networks to identify participants when referred urgently to specialist spinal centres.
- The relationship of timing of investigation and decompression to patient outcome will be limited by patient and clinician reporting of the timing of symptom onset.

For peer review only

For peer review only - http://bmjopen.bmj.com/site/about/guidelines.xhtml

INTRODUCTION

Cauda equina syndrome (CES) is a rare but potentially devastating condition caused by compression of the cauda equina nerve roots. This most commonly occurs due to a prolapsed intervertebral disc. The clinical syndrome includes any of bilateral sciatica, saddle anaesthesia, bladder, bowel, or sexual dysfunction.[1-3] The disabling nature of these symptoms causes significant medical and social morbidity and high health and social care costs. In addition, litigation related to the management of CES leads to significant medico-legal workload and costs.[1,4,5]

Due to the consequences of CES for patients and society, several groups have issued clinical guidance or standards of care for CES.[1,6-8] However, the evidence base for current clinical guidance consists of small retrospective single centre case series.[1,9,10] Even systematic reviews of outcomes in CES have included relatively few patients, with the largest including 464 patients.[9,11] Lack of a clear definition of CES has hampered comparative analysis of historic studies, and different interpretations of the available evidence have been offered.[10,12] A diagnosis of CES encompasses patients presenting with mild to severe urinary and bowel symptoms, perineal or perianal numbness, sexual function disturbance, or bilateral sciatica, and patients may also experience lower limb weakness, numbness, or unilateral sciatica.[2,3,13] Outcomes for different presentations vary, and accurate division by presentation may help to clarify the understanding of outcome studies and develop care standards appropriate to the presentation.[1,14]

Retrospective case series in the United Kingdom (UK) have identified approximately 15-31 patients per year per specialist neurosurgical or spinal centre with confirmed CES.[3,13,15,16] Published estimates of the incidence of CES are fewer than one case per 100,000 population.[17,18] However, in 2010-2011 in England, 981 surgical decompressions were performed for CES,[19] and the population was estimated at 52,234,000,[20] giving an incidence of 1.9 per 100,000. Therefore, there may be over 1000 patients managed for CES in the UK each year. Accurate data on the presentation and management of these patients would establish current management plus adherence to and feasibility of care quality statements as well as potentially informing the revision of guidance based on accurate and current data.

For peer review only - http://bmjopen.bmj.com/site/about/guidelines.xhtml

The British Neurosurgical Trainee Research Collaborative (BNTRC) has previously successfully used a network of neurosurgical trainees across the UK and Ireland to identify cases via local tertiary referral systems in conditions such as chronic subdural haematoma.[21] As CES is managed in the UK by specialist spinal services, similar case ascertainment via specialist referral systems to neurosurgical, orthopaedic, or joint spinal services provides a method of accurately identifying patients with CES during hospital admission. We propose to carry out the first national cohort study of the presentation and management of CES in the UK and establish the largest prospective series of patients with CES. This will provide data on CES incidence, epidemiology, presentation, management, and outcomes. This will inform the development of clinical guidance and identify areas for future research in CES.

This prospective observational cohort study aims to:

- Identify the number of cases of CES in the UK in all collaborating centres
- Describe the presenting symptoms and signs in patients with CES
- Describe the pathways of presentation to specialist spinal services for patients with CES in the UK
- Describe the type, timing, and findings of investigations in patients with CES
- Describe the medical and surgical management of CES
- Compare current practice to standards of care for CES
- Describe clinical outcomes for patients with CES using validated patient reported outcome measures, stratified by presentation, investigation findings, and management
- Demonstrate the ability of neurosurgical and orthopaedic surgical trainee networks to collaborate successfully on a prospective cohort study

METHODS AND ANALYSIS

Understanding Cauda Equina Syndrome (UCES) is a prospective cohort study of patients with confirmed CES managed at specialist spinal centres in the UK. Cases will be identified by neurosurgical or orthopaedic trainees in each specialist centre through daily screening of tertiary referrals and admissions to specialist spinal services. All patients managed as CES by the treating team will be included in this study. CES will be divided into CES suspected (CESS), CES incomplete (CESI), and CES retention (CESR).

For peer review only - http://bmjopen.bmj.com/site/about/guidelines.xhtml

Data regarding timing and type of symptom onset, referral, investigation, management, and outcome will be recorded anonymously on a secure database by the local trainee investigator during the patient's hospital admission and after discharge. Patient consent will be sought for the use of their data and patients will be asked to complete patient reported outcome measures representing their condition before surgery and up to one year after surgery. Imaging at presentation will also be collected. This data will be compared with care quality statements and published outcome data for CES. This is an observational study. No changes to routine patient care will occur during this study.

Participant Selection

The study will recruit for one year. Cases will be identified from admissions to spinal units between 1st June 2018 until 31st May 2019. The last one year follow up assessments will be sent to participants on 31st May 2020.

For inclusion in this study, the patient must:

- be over 18 years old;
- be admitted to a specialist spinal service in the UK between 1st June 2018 and 31st May 2019;
- have capacity to provide informed consent for participation in this study; and
- have a diagnosis of clinical CES and structural compression of the cauda equina on imaging as determined by the treating clinician.
    - Clinical CES includes any of: altered saddle sensation; bladder dysfunction; bowel dysfunction; sexual dysfunction; or bilateral sciatica. This should be associated with radiological compression of the cauda equina. The cauda equina compression can be due to any cause, including, but not limited to, disc, tumour, infection, etc.

There is no upper age limit as we aim to establish the demographics of those presenting with CES.

The exclusion criteria are:

- Children under 18 years old.

For peer review only - http://bmjopen.bmj.com/site/about/guidelines.xhtml

- Patients undergoing emergent decompression for unilateral motor or sensory symptoms (such as foot drop), without clinical evidence of CES.
- Patients referred with suspected CES where the diagnosis is not confirmed, for example patients with the clinical symptoms and signs of CES without radiological evidence of cauda equina compression.
- Patients not admitted to participating spinal centres in the UK.
- Patients admitted to a participating spinal centre before 1st June 2018 or after 31st May 2019.
- Patients who are unable to provide informed consent for participation in this study.

Capture-recapture methods will be used to ensure complete case ascertainment. In December 2018, June 2019, and December 2019 all local investigators will check their case ascertainment by asking their local coding departments for all discharges coded as CES using the diagnostic code ICD-10 G83.4. Any additional patients identified through this method that meet the inclusion criteria will be invited to participate.

Data Collection

Data relating to presentation, hospital admission, investigations, and follow up will be collected by the local trainee investigator. Data will be collected from the patient's notes, through routine interaction with the patient as part of clinical care, and through interaction with other staff members caring for the patient. All clinical and demographic information collected for this study by the local investigators is collected routinely. No extra assessments will be performed.

Study participants who have consented to participate will also be asked to fill out details about their patient journey, their symptoms, patient reported outcome measures, and service usage. These will be collected electronically anonymously via the electronic database and linked to the patient record. Patient reported outcome measures will include visual analogue scores for back and leg pain, the Oswestry Disability Index,[22] the neurogenic bowel dysfunction score,[23] the short form incontinence questionnaire,[24] and the Arizona sexual experiences scale.[25]

For peer review only - http://bmjopen.bmj.com/site/about/guidelines.xhtml

All patients who are eligible for inclusion in the study will have basic anonymous clinical data collected as part of the screening log to establish participation rates and incidence at each centre. This will allow accurate assessment of the incidence of CES. Patients who do not wish to participate in the study will not be contacted further for the completion of patient reported outcome measures.

The timing and type of clinician reported and patient reported data that will be collected for UCES is shown in Figure 1: Study Flow Diagram.

Clinician entered data will be entered directly into the database using the participant's unique study number. Imaging will be reviewed on local PACS systems and transferred to the study team for review. Participant questionnaires will be sent out by email using unique links for each participant. If participants do not have an email address or prefer to fill out questionnaires on paper, paper or telephone versions of the questionnaires will be used. If participants do not respond to the email invitations, they will be contacted to find out whether they wish to continue with the study and to complete the questionnaires when willing. Where patient data is routinely entered into spinal databases, surgical and outcome data from those databases will be linked anonymously to the patient record by the clinical team using the patient's unique identifier for that database or registry.

Data Analysis

This study aims to establish the number of patients presenting with CES in the UK over one year. We expect approximately 20 patients per spinal centre per year depending on the population served, and a total of approximately 600-1000 patients in one year across the UK. The incidence of CES will be established based on the number of patients identified at each unit and the catchment population of that unit. If all units in the UK participate, incidence will be calculated based on UK population estimates. Incidence will be calculated from all patients identified as being eligible for the study from referral screening and local coding departments even if they do not consent for further participation.

A descriptive analysis of the clinical and demographic characteristics of presenting symptoms, signs, and outcomes of patients with CES will be performed. This will be determined from both clinician reported and patient reported data. CES incidence

For peer review only - http://bmjopen.bmj.com/site/about/guidelines.xhtml

and characteristics will be broken down into the categories of CESS, CESI, and CESR based on the clinical data. The categorical and quantitative findings on imaging will also be described. Methods of patient presentation to specialist services will be described. Type, timings, and findings of investigations in patients presenting via different routes will be compared. The investigation and management of patients with CES will be described and compared to that laid out in current care quality standards. Proportions meeting the standards will be reported. Patient outcomes will be assessed and analysed using both clinician and patient reported outcome measures at six months and one year. Patient outcomes will be stratified by demographics, presenting features, timing and findings of investigations, and timing and type of surgery. Patient usage of healthcare services over the year following diagnosis and management of CES will be assessed using both patient reported service usage and electronic records.

ETHICS AND DISSEMINATION

Patient Consent

Once patients have been identified as being eligible to participate in the study, they will be asked by a member of their clinical team whether they would be willing to receive further information about the study. For the majority of patients this will occur during their admission to the spinal unit, and the approach will be made by a member of ward medical or nursing staff. Once verbal consent has been gained to give further information about the study, patients will be provided with the information leaflet for the study. Patients who indicate that they are happy to have further discussions regarding the study will be visited in hospital by a member of their clinical team to complete the written consent process. The person undertaking written consent will be adequately trained to do so, and have a good knowledge of the study protocol, aims, and processes. The participant will be informed about and consent to their medical records being inspected by regulatory authorities and representatives of the sponsor. Both the participant and the person undertaking consent will sign and date the informed consent form to confirm that consent has been obtained. The participant will receive a copy of this document and a copy will be filed in the Investigator Site File.

Decompression surgery for CES takes place as an emergency, and admissions occur at all times of day and night throughout the week and weekend. Following decompression the length of stay in hospital wards may be as short as one to two

For peer review only - http://bmjopen.bmj.com/site/about/guidelines.xhtml

days, or may be longer than a week when there are ongoing bladder or bowel problems. All patients will be given adequate time to read the information leaflet with a minimum time period of six hours. Some patients will be discharged prior to being identified as being eligible for the study. These patients will be contacted by telephone by a member of the clinical team and asked if they would be willing to receive information about the study by post or email. If they agree, the information leaflet and consent form will be sent to them, and they will be re-contacted to go through the consent process over the telephone at least 24 hours after receiving the information.

When participants prefer to fill out paper questionnaires or do not respond to the email link, their contact details (name, address, telephone number) will be passed to the central study team at NHS Lothian using the NHS email system with the consent of the patient. The central study team will contact the participants to find out whether they still wish to take part in the study. Those who wish to continue with the study will be sent the questionnaires by email, by post, or they can be completed over the telephone with a member of the central study team depending on the preference of the participant. If participants do not wish to continue with the study, they will not be contacted further.

Participants are free to withdraw from the study at any point. If withdrawal occurs, the primary reason for withdrawal will be documented in the participant's electronic case report form. The participant will not be contacted any further for outcome measures but their basic anonymous clinical details will be retained to allow accurate epidemiological assessment of the incidence of CES. If a patient loses capacity to consent for ongoing participation during the course of the study, the data they have already submitted or has already been submitted by their clinical team with their consent will continue to be used in the study, but they will not be contacted with further questionnaires.

Data Protection

All Investigators and study site staff involved with this study will comply with the requirements of the Data Protection Act 1998 with regard to the collection, storage, processing and disclosure of personal information and will uphold the Act's core principles. Access to collated participant data will be restricted to individuals from the

For peer review only - http://bmjopen.bmj.com/site/about/guidelines.xhtml
For peer review only

research team treating the participants, representatives of the sponsor and representatives of regulatory authorities. Computers used to collate the data will have limited access measures via user names and passwords. Published results will not contain any personal data that could allow identification of individual participants.

All clinical details will be entered into a database hosted by Castor EDC. Castor EDC complies with all applicable laws and regulations: Good Clinical Practice (GCP), European Union (EU) Annex 11, and the European Data Protection Directive. Clinician entered data will be entered directly into the database using the participant's unique study number. The clinical team can only view the records of patients from their own centre. Once a participant has consented for their email address to be stored, this will be entered into the Castor database by the local clinical team. The email address field is stored securely and is encrypted and cannot be viewed by anyone outside of the patient's local centre.

All local investigators will store a copy of the link between the patient's unique study number and their contact details, National Health Service (NHS) number, hospital number, Community Health Index (CHI) number, unique identifiers for spinal databases or registries, or other identifying details on a secure password protected NHS computer. Consent forms and paper completed questionnaires will be stored securely in a locked NHS office. No identifying information will be entered into the secure database except the email address.

All identifiable scans will be stored and transferred within the NHS PACS network. Only anonymised scans will be processed outside the NHS PACS network. Anonymised imaging data will be labelled only with the study number and stored on anonymised CDs or on encrypted hard drives.

Data Retention

All study documentation will be kept for a minimum of 5 years from the end of the study. When the minimum retention period has elapsed, study documentation will not be destroyed without permission from the sponsor. The end of the study is 18 months after the enrolment of the last participant.

For peer review only - http://bmjopen.bmj.com/site/about/guidelines.xhtml

Insurance and Indemnity

Sites participating in the study will be liable for clinical negligence and other negligent harm to individuals taking part in the study and covered by the duty of care owed to them by the sites concerned. The sponsor requires individual sites participating in the study to arrange for their own insurance or indemnity in respect of these liabilities. Sites which are part of the United Kingdom's National Health Service will have the benefit of NHS Indemnity.

Ethical Review

The study will be conducted in accordance with the principles of the International Conference on Harmonisation Tripartite Guideline for Good Clinical Practice (GCP). All researchers are encouraged to undertake GCP training in order to understand the principles of GCP. However, this is not a mandatory requirement. GCP training status for all investigators should be indicated in their respective CVs.

UCES received a favourable ethical opinion from the South East Scotland Research Ethics Committee (REC) 02 (reference 18/SS/0047, IRAS reference: 233515, sponsor reference: AC18017). Local management approvals must be in place at each site prior to recruitment of patients to this study. This study is registered with ClinicalTrials.gov (Record 160318) and is pending registration with ISRCTN. The most recent version of the protocol will be available on the website of the BNTRC at www.bntrc.org.uk. This study is sponsored by NHS Lothian.

Peer Review

The concept for this study was selected by a panel of judges in an open competition for support from the BNTRC. The protocol has been reviewed and approved by the steering committee for this study and reviewed by the British Orthopaedic Trainees' Association, the British Association of Spine Surgeons, and the BNTRC committee.

Publication

Ownership of the complete dataset arising from this study resides with the steering committee and the BNTRC. On completion of the study, the data will be analysed and tabulated, and a report will be prepared. A summary report of the study will be provided to the REC within one year of the end of the study. Local data collected as part of this study belongs to the local team collecting that data. The study report will

For peer review only - http://bmjopen.bmj.com/site/about/guidelines.xhtml

be used for publication and presentation at scientific meetings. Summaries of results will also be made available to local investigators. Following the initial analysis and publication, study data will be made available to those who submit successful peer-reviewed proposals for use of the data to the steering committee via the BNTRC.

All local investigators who enter data for at least one case will be named as contributors on publications arising from this study and will receive a certificate of collaboration in this study. Authorship of publications arising from this study will be determined in accordance with the guidelines of the International Committee of Medical Journal Editors (ICMJE).[26]

For peer review only

For peer review only - http://bmjopen.bmj.com/site/about/guidelines.xhtml

REFERENCES

1. Todd NV, Dickson RA. Standards of care in cauda equina syndrome. *BMJ* 2016;30(5):518-22. doi: 10.1080/02688697.2016.1187254
2. Fraser S, Roberts L, Murphy E. Cauda equina syndrome: a literature review of its definition and clinical presentation. *Arch Phys Med Rehabil* 2009;90(11):1964-8. doi: 10.1016/j.apmr.2009.03.021
3. Balasubramanian K, Kalsi P, Greenough CG, et al. Reliability of clinical assessment in diagnosing cauda equina syndrome. *Br J Neurosurg* 2010;24(4):383-6. doi: 10.3109/02688697.2010.505987
4. Todd NV. Causes and outcomes of cauda equina syndrome in medico-legal practice: a single neurosurgical experience of 40 consecutive cases. *Br J Neurosurg* 2011;25(4):503-8. doi: 10.3109/02688697.2010.550344
5. Quraishi NA, Hammett TC, Todd DB, et al. Malpractice litigation and the spine: the NHS perspective on 235 successful claims in England. *Eur Spine J* 2012;21 Suppl 2:S196-9. doi: 10.1007/s00586-012-2203-5
6. Germon T, Ahuja S, Casey AT, et al. British Association of Spine Surgeons standards of care for cauda equina syndrome. *Spine J* 2015;15(3 Suppl):S2-4. doi: 10.1016/j.spinee.2015.01.006
7. Society of British Neurological Surgeons. Standards of Care for Established and Suspected Cauda Equina Syndrome, 2009, version 4.
8. Society of British Neurological Surgeons. SBNS Care Quality Statement. 2015.
9. Ahn UM, Ahn NU, Buchowski JM, et al. Cauda equina syndrome secondary to lumbar disc herniation: a meta-analysis of surgical outcomes. *Spine* 2000;25(12):1515-22.
10. Kohles SS, Kohles DA, Karp AP, et al. Time-dependent surgical outcomes following cauda equina syndrome diagnosis: comments on a meta-analysis. *Spine* 2004;29(11):1281-7.
11. Korse NS, Jacobs WC, Elzevier HW, et al. Complaints of micturition, defecation and sexual function in cauda equina syndrome due to lumbar disk herniation: a systematic review. *Eur Spine J* 2013;22(5):1019-29. doi: 10.1007/s00586-012-2601-8 .
12. Gardner A, Gardner E, Morley T. Cauda equina syndrome: a review of the current clinical and medico-legal position. *Eur Spine J* 2011;20(5):690-7. doi: 10.1007/s00586-010-1668-3
13. Bell DA, Collie D, Statham PF. Cauda equina syndrome: what is the correlation between clinical assessment and MRI scanning? *Br J Neurosurg* 2007;21(2):201-3. doi: 10.1080/02688690701317144
14. Gleave JR, Macfarlane R. Cauda equina syndrome: what is the relationship between timing of surgery and outcome? *Br J Neurosurg* 2002;16(4):325-8.
15. Srikandarajah N, Boissaud-Cooke MA, Clark S, et al. Does early surgical decompression in cauda equina syndrome improve bladder outcome? *Spine* 2015;40(8):580-3. doi: 10.1097/brs.0000000000000813
16. Crocker M, Fraser G, Boyd E, et al. The value of interhospital transfer and emergency MRI for suspected cauda equina syndrome: a 2-year retrospective study. *Ann R Coll Surg Engl* 2008;90(6):513-6. doi: 10.1308/003588408x301154
17. Hurme M, Alaranta H, Torma T, et al. Operated lumbar disc herniation: epidemiological aspects. *Ann Chir Gynaecol* 1983;72(1):33-6.
18. Podnar S. Epidemiology of cauda equina and conus medullaris lesions. *Muscle Nerve* 2007;35(4):529-31. doi: https://dx.doi.org/10.1002/mus.20696
19. The National Spinal Taskforce. Commissioning Spinal Services - Getting The Service Back On Track, January 2013.

For peer review only - http://bmjopen.bmj.com/site/about/guidelines.xhtml

20. Office for National Statistics. Annual Mid-year Population Estimates, 2010. Statistical Bulletin, 30 June 2011.
21. Brennan PM, Kolias AG, Joannides AJ, et al. The management and outcome for patients with chronic subdural hematoma: a prospective, multicenter, observational cohort study in the United Kingdom. *J Neurosurg* 2016:1-8. doi: 10.3171/2016.8.jns16134
22. Fairbank JC, Pynsent PB. The Oswestry Disability Index. *Spine* 2000;25(22):2940-52
23. Krogh K, Christensen P, Sabroe S, et al. Neurogenic bowel dysfunction score. *Spinal Cord* 2006;44(10):625-31. doi: 10.1038/sj.sc.3101887
24. McGrother CW, Donaldson MM, Shaw C, et al. Storage symptoms of the bladder: prevalence, incidence and need for services in the UK. *BJU Int* 2004;93(6):763-9. doi: 10.1111/j.1464-410X.2003.04721.x
25. McGahuey CA, Gelenberg AJ, Laukes CA, et al. The Arizona Sexual Experience Scale (ASEX): reliability and validity. *J Sex Marital Ther* 2000;26(1):25-40.
26. International Committee of Medical Journal Editors. Recommendations for the Conduct, Reporting, Editing and Publication of Scholarly Work in Medical Journals.

For peer review only

For peer review only - http://bmjopen.bmj.com/site/about/guidelines.xhtml

AUTHORS' CONTRIBUTIONS

JW, IH, SP, and AABJ contributed to the conception of the study, design of the study, writing of the protocol, revision of the protocol, and approving the final version.
NS, MP, HR, AKD, PS, NE, and PFXS contributed to the design of the study, revision of the protocol, and approving the final version

FUNDING STATEMENT

This research received no specific grant from any funding agency in the public, commercial or not-for-profit sectors.

COMPETING INTERESTS STATEMENT

JW reports grants from the Wellcome Trust during this study. IH reports grants from the Association of British Neurologists/Patrick Berthoud Charitable Trust during this study. AABJ, SP, NS, MP, HR, AKD, PS, NE, and PFXS have nothing to disclose.

ACKNOWLEDGEMENTS

We would like to acknowledge the guidance and support in developing this protocol from the study steering committee (Simon Clark, Andreas Demetriades, Gareth Dobson, Niall Eames, Ingrid Hoeritzauer, Aimun Jamjoom, Andraay Leung, Michael Poon, Manjunath Prasad, Savva Pronin, Phil Sell, Nisaharan Srikandarajah, Patrick Statham, Stacey Thomson, Martin Wilby, Julie Woodfield), the British Association of Spinal Surgeons (Mike Hutton, David Cumming, Jake Timothy, Andraay Leung, Chrishan Thakar, Tim Germon), the British Orthopaedic Trainees Assocation (Rory Morrison), the British Neurosurgical Trainee Research Collaborative (Ellie Edlman, Ruichong Ma, Michael Poon), and the sponsor NHS Lothian (Kenneth Scott).

FIGURE LEGENDS

Figure 1: Study Flow Diagram. Time points for patient reported and clinician reported data collection in UCES.

For peer review only - http://bmjopen.bmj.com/site/about/guidelines.xhtml

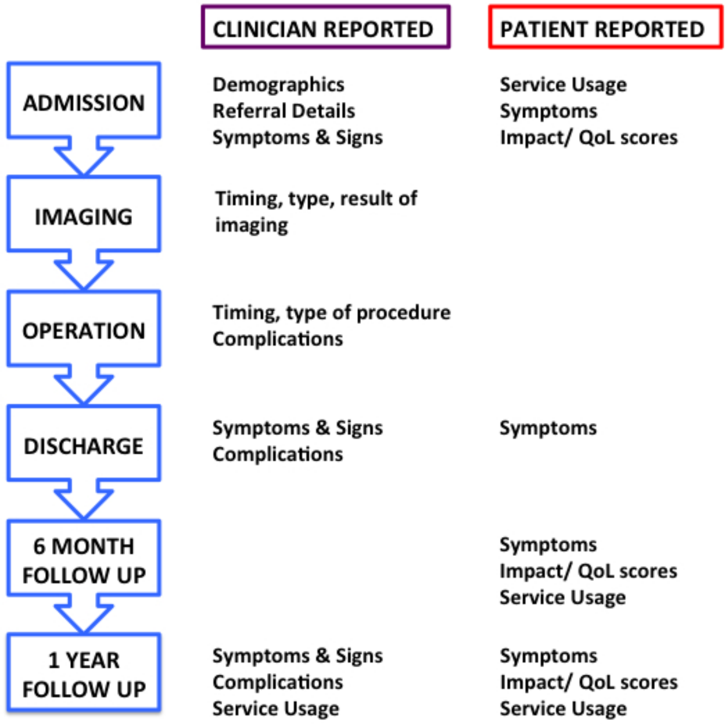

Figure 1: Study Flow Diagram. Time points for patient reported and clinician reported data collection in UCES.

BMJ Open

Understanding Cauda Equina Syndrome: protocol for a United Kingdom multi-centre prospective observational cohort study

Journal:	*BMJ Open*
Manuscript ID	bmjopen-2018-025230.R1
Article Type:	Protocol
Date Submitted by the Author:	01-Oct-2018

Complete List of Authors:	Woodfield, Julie; Western General Hospital, Department for Clinical Neurosciences; The University of Edinburgh, Centre for Clinical Brain Sciences<br>Hoeritzauer, Ingrid; Western General Hospital, Department for Clinical Neurosciences; The University of Edinburgh, Centre for Clinical Brain Sciences<br>Jamjoom, Aimun; The University of Edinburgh, Centre for Clinical Brain Sciences; Aberdeen Royal Infirmary, Department of Neurosurgery<br>Pronin, Savva; The University of Edinburgh, Centre for Clinical Brain Sciences<br>Srikandarajah, Nisaharan; Walton Centre NHS Foundation Trust, Department of Neurosurgery<br>Poon, Michael; Western General Hospital, Department for Clinical Neurosciences<br>Roy, Holly; Derriford Hospital, South West Neurosurgery Centre<br>Demetriades, Andreas; Western General Hospital, Department for Clinical Neurosciences<br>Sell, Philip; Queens Medical Centre, Centre for Spinal Studies and Surgery<br>Eames, Niall; Royal Victoria Hospital, Trauma and Orthopaedics<br>Statham, Patrick; Western General Hospital, Department for Clinical Neurosciences<br>Research Collaborative, British Neurosurgical Trainee ; British Neurosurgical Trainee Research Collaborative, British Neurosurgical Trainee Research Collaborative

**Primary Subject Heading**:	Surgery
Secondary Subject Heading:	Health services research
Keywords:	cauda equina syndrome, spinal surgery, cohort study, outcomes

SCHOLARONE™
Manuscripts

TITLE

Understanding Cauda Equina Syndrome: protocol for a United Kingdom multi-centre prospective observational cohort study

AUTHORS

Julie Woodfield[1,2], Ingrid Hoeritzauer[1,2], Aimun AB Jamjoom[2,3], Savva Pronin[2], Nisaharan Srikandarajah[4], Michael Poon[1], Holly Roy[5], Andreas K Demetriades[1], Phil Sell[6], Niall Eames[7], Patrick Statham[1], British Neurosurgical Trainee Research Collaborative (BNTRC)

1. Western General Hospital, Department of Clinical Neurosciences, Edinburgh, UK
2. The University of Edinburgh, Centre for Clinical Brain Sciences, Edinburgh, UK
3. Aberdeen Royal Infirmary, Department of Neurosurgery, Aberdeen, UK
4. Walton Centre NHS Foundation Trust, Department of Neurosurgery, Liverpool, UK
5. Derriford Hospital, South West Neurosurgery Centre, Plymouth, UK
6. Queens Medical Centre, Centre for Spinal Studies and Surgery, Nottingham, UK
7. Royal Victoria Hospital, Trauma and Orthopaedics, Belfast, UK

Corresponding Author:

Name: Julie Woodfield, Neurosurgery Specialty Trainee

Address: Department of Clinical Neurosciences, Western General Hospital, Crewe Road South, Edinburgh, United Kingdom. EH4 2XU

Email: julie.woodfield@ed.ac.uk

Telephone: 0131 537 1000

WORD COUNT

3335

For peer review only

For peer review only - http://bmjopen.bmj.com/site/about/guidelines.xhtml

ABSTRACT

Introduction

Cauda equina syndrome (CES) is a potentially devastating condition caused by compression of the cauda equina nerve roots. This can result in bowel, bladder and sexual dysfunction plus lower limb weakness, numbness, and pain. CES occurs infrequently but has serious potential morbidity and medico-legal consequences. This study aims to identify and describe the presentation and management of patients with CES in the United Kingdom (UK).

Methods and Analysis

Understanding Cauda Equina Syndrome (UCES) is a prospective, collaborative, multicentre cohort study of adult patients with confirmed CES managed at specialist spinal centres in the UK. Participants will be identified using neurosurgical and orthopaedic trainee networks to screen referrals to spinal centres. Details of presentation, investigations, management and service usage will be recorded. Both patient and clinician reported outcome measures will be assessed for one year after surgery. This will establish the incidence of CES, current investigation and management practices, and adherence to national standards of care. Outcomes will be stratified by clinical presentation and patient management. Accurate, up to date information about the presentation, management, and outcome of patients with cauda equina syndrome will inform standards of service design and delivery for this important but infrequent condition.

Ethics and Dissemination

UCES received a favourable ethical opinion from the South East Scotland Research Ethics Committee 02 (Reference: 18/SS/0047; IRAS ID: 233515). All spinal centres managing patients with CES in the UK will be encouraged to participate in UCES. Study results will be published in medical journals and shared with local participating sites.

Registration Details

UCES is sponsored by NHS Lothian (Reference: AC18017). UCES is registered at ClinicalTrials.gov (160318) and ISRCTN (ISRCTN16828522).

For peer review only

For peer review only - http://bmjopen.bmj.com/site/about/guidelines.xhtml

KEYWORDS

Cauda Equina Sndrome, cohort study, outcomes, spinal surgery

ARTICLE SUMMARY

Strengths and Limitations of this Study

- This UK wide study will be the largest prospectively established cohort of patients with CES.
- The collection of detailed clinical data will describe the range of presentations treated as CES in the UK in current practice and allow stratification of findings by clinical presentation.
- Validated outcome measures will be used to assess pain, disability, and bladder, bowel, and sexual function one year after treatment.
- Participant identification and recruitment will be efficiently carried out using trainee research networks to identify participants when referred urgently to specialist spinal centres.
- The relationship of timing of investigation and decompression to patient outcome will be limited by patient and clinician reporting of the timing of symptom onset.

For peer review only - http://bmjopen.bmj.com/site/about/guidelines.xhtml
For peer review only

INTRODUCTION

Cauda equina syndrome (CES) is a rare but potentially devastating condition caused by compression of the cauda equina nerve roots. This most commonly occurs due to a prolapsed intervertebral disc. The clinical syndrome includes any of bilateral sciatica, saddle anaesthesia, bladder, bowel, or sexual dysfunction.[1-3] The disabling nature of these symptoms causes significant medical and social morbidity and high health and social care costs. In addition, litigation related to the management of CES leads to significant medico-legal workload and costs.[1,4,5]

Due to the consequences of CES for patients and society, several groups have issued clinical guidance or standards of care for CES.[1,6-8] However, the evidence base for current clinical guidance consists of small retrospective single centre case series.[1,9,10] Even systematic reviews of outcomes in CES have included relatively few patients, with the largest including 464 patients.[9,11] Lack of a clear definition of CES has hampered comparative analysis of historic studies, and different interpretations of the available evidence have been offered.[10,12] A diagnosis of CES encompasses patients presenting with mild to severe urinary and bowel symptoms, perineal or perianal numbness, sexual function disturbance, or bilateral sciatica, and patients may also experience lower limb weakness, numbness, or unilateral sciatica.[2,3,13] Outcomes for different presentations vary, and accurate division by presentation may help to clarify the understanding of outcome studies and develop care standards appropriate to the presentation.[1,14]

Retrospective case series in the United Kingdom (UK) have identified approximately 15-31 patients per year per specialist neurosurgical or spinal centre with confirmed CES.[3,13,15,16] Published estimates of the incidence of CES are fewer than one case per 100,000 population.[17,18] However, in 2010-2011 in England, 981 surgical decompressions were performed for CES,[19] and the population was estimated at 52,234,000,[20] giving an incidence of 1.9 per 100,000. Therefore, there may be over 1000 patients managed for CES in the UK each year. Accurate data on the presentation and management of these patients would establish current management plus adherence to and feasibility of care quality statements as well as potentially informing the revision of guidance based on accurate and current data.

For peer review only - http://bmjopen.bmj.com/site/about/guidelines.xhtml

The British Neurosurgical Trainee Research Collaborative (BNTRC) has previously successfully used a network of neurosurgical trainees across the UK and Ireland to identify cases via local tertiary referral systems in conditions such as chronic subdural haematoma.[21] As CES is managed in the UK by specialist spinal services, similar case ascertainment via specialist referral systems to neurosurgical, orthopaedic, or joint spinal services provides a method of accurately identifying patients with CES during hospital admission. We propose to carry out the first national cohort study of the presentation and management of CES in the UK and establish the largest prospective series of patients with CES. This will provide data on CES incidence, epidemiology, presentation, management, and outcomes. This will inform the development of clinical guidance and identify areas for future research in CES.

This prospective observational cohort study aims to:

- Identify the number of cases of CES in the UK in all collaborating centres
- Describe the presenting symptoms and signs in patients with CES
- Describe the pathways of presentation to specialist spinal services for patients with CES in the UK
- Describe the type, timing, and findings of investigations in patients with CES
- Describe the medical and surgical management of CES
- Compare current practice to standards of care for CES
- Describe clinical outcomes for patients with CES using validated patient reported outcome measures, stratified by presentation, investigation findings, and management
- Demonstrate the ability of neurosurgical and orthopaedic surgical trainee networks to collaborate successfully on a prospective cohort study

METHODS AND ANALYSIS

Understanding Cauda Equina Syndrome (UCES) is a prospective cohort study of patients with confirmed CES managed at specialist spinal centres in the UK. Cases will be identified by neurosurgical or orthopaedic trainees in each specialist centre through daily screening of tertiary referrals and admissions to specialist spinal services. All patients managed as CES by the treating team will be included in this study.

For peer review only - http://bmjopen.bmj.com/site/about/guidelines.xhtml

Data regarding timing and type of symptom onset, referral, investigation, management, and outcome will be recorded anonymously on a secure database by the local trainee investigator during the patient's hospital admission and after discharge. Patient consent will be sought for the use of their data and patients will be asked to complete patient reported outcome measures representing their condition before surgery and up to one year after surgery. Imaging at presentation will also be collected. This data will be compared with care quality statements and published outcome data for CES. This is an observational study. No changes to routine patient care will occur during this study.

Participant Selection

The study will recruit for one year. Cases will be identified from admissions to spinal units between 1st June 2018 until 31st May 2019. The last one year follow up assessments will be sent to participants on 31st May 2020.

For inclusion in this study, the patient must:

- be over 18 years old;
- be admitted to a specialist spinal service in the UK between 1st June 2018 and 31st May 2019;
- have capacity to provide informed consent for participation in this study; and
- have a diagnosis of clinical CES and structural compression of the cauda equina on imaging as determined by the treating clinician.
    - Clinical CES includes any of: altered saddle sensation; bladder dysfunction; bowel dysfunction; sexual dysfunction; or bilateral sciatica. This should be associated with radiological compression of the cauda equina. The cauda equina compression can be due to any cause, including, but not limited to, disc, tumour, infection, etc.

There is no upper age limit as we aim to establish the demographics of those presenting with CES.

The exclusion criteria are:

- Children under 18 years old.
- Patients undergoing emergent decompression for unilateral motor or sensory symptoms (such as foot drop), without clinical evidence of CES.

For peer review only - http://bmjopen.bmj.com/site/about/guidelines.xhtml

- Patients referred with suspected CES where the diagnosis is not confirmed, for example patients with the clinical symptoms and signs of CES without radiological evidence of cauda equina compression.
- Patients not admitted to participating spinal centres in the UK.
- Patients admitted to a participating spinal centre before 1st June 2018 or after 31st May 2019.
- Patients who are unable to provide informed consent for participation in this study.

Capture-recapture methods will be used to ensure complete case ascertainment. In December 2018, June 2019, and December 2019 all local investigators will check their case ascertainment by asking their local coding departments for all discharges coded as CES using the diagnostic code ICD-10 G83.4. Any additional patients identified through this method that meet the inclusion criteria will be invited to participate.

Data Collection

Data relating to presentation, hospital admission, investigations, and follow up will be collected by the local trainee investigator. Data will be collected from the patient's notes, through routine interaction with the patient as part of clinical care, and through interaction with other staff members caring for the patient. All clinical and demographic information collected for this study by the local investigators is collected routinely. No extra assessments will be performed.

Study participants who have consented to participate will also be asked to fill out details about their patient journey, their symptoms, patient reported outcome measures, and service usage. These will be collected electronically anonymously via the electronic database and linked to the patient record. Patient reported outcome measures will include visual analogue scores for back and leg pain plus the relevant sections of the Oswestry Disability Index,[22] the neurogenic bowel dysfunction score,[23] the short form incontinence questionnaire,[24] and the Arizona sexual experiences scale.[25]

All patients who are eligible for inclusion in the study will have basic anonymous clinical data collected as part of the screening log to establish participation rates and

For peer review only - http://bmjopen.bmj.com/site/about/guidelines.xhtml

incidence at each centre. This will allow accurate assessment of the incidence of CES. Patients who do not wish to participate in the study will not be contacted further for the completion of patient reported outcome measures.

The timing and type of clinician reported and patient reported data that will be collected for UCES is shown in Figure 1: Study Flow Diagram.

Clinician entered data will be entered directly into the database using the participant's unique study number. Imaging will be reviewed on local PACS systems and transferred to the study team for review. Participant questionnaires will be sent out by email using unique links for each participant. If participants do not have an email address or prefer to fill out questionnaires on paper, paper or telephone versions of the questionnaires will be used. If participants do not respond to the email invitations, they will be contacted to find out whether they wish to continue with the study and to complete the questionnaires when willing. Where patient data is routinely entered into spinal databases, surgical and outcome data from those databases will be linked anonymously to the patient record by the clinical team using the patient's unique identifier for that database or registry.

Data Analysis

This study aims to establish the number of patients presenting with CES in the UK over one year. We expect approximately 20 patients per spinal centre per year depending on the population served, and a total of approximately 600-1000 patients in one year across the UK. The incidence of CES will be established based on the number of patients identified at each unit and the catchment population of that unit. If all units in the UK participate, incidence will be calculated based on UK population estimates. Incidence will be calculated from all patients identified as being eligible for the study from referral screening and local coding departments even if they do not consent for further participation.

A descriptive analysis of the clinical and demographic characteristics of presenting symptoms, signs, and outcomes of patients with CES will be performed. This will be determined from both clinician reported and patient reported data. CES incidence and characteristics will be broken down into categories such as suspected (CESS), incomplete (CESI), with retention (CESR), and early (CESE) based on the clinical

For peer review only - http://bmjopen.bmj.com/site/about/guidelines.xhtml

data. The categorical and quantitative findings on imaging will also be described. Methods of patient presentation to specialist services will be described. Type, timings, and findings of investigations in patients presenting via different routes will be compared. The investigation and management of patients with CES will be described and compared to that laid out in current care quality standards. Proportions meeting the standards will be reported. Patient outcomes will be assessed and analysed using both clinician and patient reported outcome measures at six months and one year. Patient outcomes will be stratified by demographics, presenting features, causative pathology, timing and findings of investigations, and timing and type of surgery. Patient usage of healthcare services over the year following diagnosis and management of CES will be assessed using both patient reported service usage and electronic records.

Patient and Public Involvement

The design and aims of this study were discussed with current patients being investigated for CES and those who had previously been treated for CES. Patients trialled the questionnaires and provided feedback on the questionnaires and patient information leaflet. The length and content of the questionnaires and information leaflet were altered in response to patient feedback. All participants will receive a summary of the results of this study. Patients are not involved in recruitment to this study as this occurs during or after emergency admission to hospital with CES.

ETHICS AND DISSEMINATION

Patient Consent

Once patients have been identified as being eligible to participate in the study, they will be asked by a member of their clinical team whether they would be willing to receive further information about the study. For the majority of patients this will occur during their admission to the spinal unit, and the approach will be made by a member of ward medical or nursing staff. Once verbal consent has been gained to give further information about the study, patients will be provided with the information leaflet for the study. Patients who indicate that they are happy to have further discussions regarding the study will be visited in hospital by a member of their clinical team to complete the written consent process. The person undertaking written consent will be adequately trained to do so, and have a good knowledge of the study protocol, aims, and processes. The participant will be informed about and consent to their medical

For peer review only

For peer review only - http://bmjopen.bmj.com/site/about/guidelines.xhtml

records being inspected by regulatory authorities and representatives of the sponsor. Both the participant and the person undertaking consent will sign and date the informed consent form to confirm that consent has been obtained. The participant will receive a copy of this document and a copy will be filed in the Investigator Site File.

Decompression surgery for CES takes place as an emergency, and admissions occur at all times of day and night throughout the week and weekend. Following decompression the length of stay in hospital wards may be as short as one to two days, or may be longer than a week when there are ongoing bladder or bowel problems. All patients will be given adequate time to read the information leaflet with a minimum time period of six hours. Some patients will be discharged prior to being identified as being eligible for the study. These patients will be contacted by telephone by a member of the clinical team and asked if they would be willing to receive information about the study by post or email. If they agree, the information leaflet and consent form will be sent to them, and they will be re-contacted to go through the consent process over the telephone at least 24 hours after receiving the information.

When participants prefer to fill out paper questionnaires or do not respond to the email link, their contact details (name, address, telephone number) will be passed to the central study team at NHS Lothian using the NHS email system with the consent of the patient. The central study team will contact the participants to find out whether they still wish to take part in the study. Those who wish to continue with the study will be sent the questionnaires by email, by post, or they can be completed over the telephone with a member of the central study team depending on the preference of the participant. If participants do not wish to continue with the study, they will not be contacted further.

Participants are free to withdraw from the study at any point. If withdrawal occurs, the primary reason for withdrawal will be documented in the participant's electronic case report form. The participant will not be contacted any further for outcome measures but their basic anonymous clinical details will be retained to allow accurate epidemiological assessment of the incidence of CES. If a patient loses capacity to consent for ongoing participation during the course of the study, the data they have already submitted or has already been submitted by their clinical team with their

For peer review only - http://bmjopen.bmj.com/site/about/guidelines.xhtml

consent will continue to be used in the study, but they will not be contacted with further questionnaires.

Data Protection

All Investigators and study site staff involved with this study will comply with the requirements of the Data Protection Act 1998 with regard to the collection, storage, processing and disclosure of personal information and will uphold the Act's core principles. Access to collated participant data will be restricted to individuals from the research team treating the participants, representatives of the sponsor and representatives of regulatory authorities. Computers used to collate the data will have limited access measures via user names and passwords. Published results will not contain any personal data that could allow identification of individual participants.

All clinical details will be entered into a database hosted by Castor EDC. Castor EDC complies with all applicable laws and regulations: Good Clinical Practice (GCP), European Union (EU) Annex 11, and the European Data Protection Directive. Clinician entered data will be entered directly into the database using the participant's unique study number. The clinical team can only view the records of patients from their own centre. Once a participant has consented for their email address to be stored, this will be entered into the Castor database by the local clinical team. The email address field is stored securely and is encrypted and cannot be viewed by anyone outside of the patient's local centre.

All local investigators will store a copy of the link between the patient's unique study number and their contact details, National Health Service (NHS) number, hospital number, Community Health Index (CHI) number, unique identifiers for spinal databases or registries, or other identifying details on a secure password protected NHS computer. Consent forms and paper completed questionnaires will be stored securely in a locked NHS office. No identifying information will be entered into the secure database except the email address.

All identifiable scans will be stored and transferred within the NHS PACS network. Only anonymised scans will be processed outside the NHS PACS network. Anonymised imaging data will be labelled only with the study number and stored on anonymised CDs or on encrypted hard drives.

For peer review only - http://bmjopen.bmj.com/site/about/guidelines.xhtml

Data Retention

All study documentation will be kept for a minimum of 5 years from the end of the study. When the minimum retention period has elapsed, study documentation will not be destroyed without permission from the sponsor. The end of the study is 18 months after the enrolment of the last participant.

Insurance and Indemnity

Sites participating in the study will be liable for clinical negligence and other negligent harm to individuals taking part in the study and covered by the duty of care owed to them by the sites concerned. The sponsor requires individual sites participating in the study to arrange for their own insurance or indemnity in respect of these liabilities. Sites which are part of the United Kingdom's National Health Service will have the benefit of NHS Indemnity.

Ethical Review

The study will be conducted in accordance with the principles of the International Conference on Harmonisation Tripartite Guideline for Good Clinical Practice (GCP). All researchers are encouraged to undertake GCP training in order to understand the principles of GCP. However, this is not a mandatory requirement. GCP training status for all investigators should be indicated in their respective CVs.

UCES received a favourable ethical opinion from the South East Scotland Research Ethics Committee (REC) 02 (reference 18/SS/0047, IRAS reference: 233515, sponsor reference: AC18017). Local management approvals must be in place at each site prior to recruitment of patients to this study. This study is registered with ClinicalTrials.gov (160318) and at ISRCTN (ISRCTN16828522). The most recent version of the protocol will be available on the website of the BNTRC at www.bntrc.org.uk. This study is sponsored by NHS Lothian.

Peer Review

The concept for this study was selected by a panel of judges in an open competition for support from the BNTRC. The protocol has been reviewed and approved by the steering committee for this study and reviewed by the British Orthopaedic Trainees' Association, the British Association of Spine Surgeons, and the BNTRC committee.

For peer review only - http://bmjopen.bmj.com/site/about/guidelines.xhtml

Publication

Ownership of the complete dataset arising from this study resides with the steering committee and the BNTRC. On completion of the study, the data will be analysed and tabulated, and a report will be prepared. A summary report of the study will be provided to the REC within one year of the end of the study. Local data collected as part of this study belongs to the local team collecting that data. The study report will be used for publication and presentation at scientific meetings. Summaries of results will also be made available to local investigators. Following the initial analysis and publication, study data will be made available to those who submit successful peer-reviewed proposals for use of the data to the steering committee via the BNTRC. All local investigators who enter data for at least one case will be named as contributors on publications arising from this study and will receive a certificate of collaboration in this study. Authorship of publications arising from this study will be determined in accordance with the guidelines of the International Committee of Medical Journal Editors (ICMJE).[26]

For peer review only

For peer review only - http://bmjopen.bmj.com/site/about/guidelines.xhtml

REFERENCES

1. Todd NV, Dickson RA. Standards of care in cauda equina syndrome. *BMJ* 2016;30(5):518-22. doi: 10.1080/02688697.2016.1187254
2. Fraser S, Roberts L, Murphy E. Cauda equina syndrome: a literature review of its definition and clinical presentation. *Arch Phys Med Rehabil* 2009;90(11):1964-8. doi: 10.1016/j.apmr.2009.03.021
3. Balasubramanian K, Kalsi P, Greenough CG, et al. Reliability of clinical assessment in diagnosing cauda equina syndrome. *Br J Neurosurg* 2010;24(4):383-6. doi: 10.3109/02688697.2010.505987
4. Todd NV. Causes and outcomes of cauda equina syndrome in medico-legal practice: a single neurosurgical experience of 40 consecutive cases. *Br J Neurosurg* 2011;25(4):503-8. doi: 10.3109/02688697.2010.550344
5. Quraishi NA, Hammett TC, Todd DB, et al. Malpractice litigation and the spine: the NHS perspective on 235 successful claims in England. *Eur Spine J* 2012;21 Suppl 2:S196-9. doi: 10.1007/s00586-012-2203-5
6. Germon T, Ahuja S, Casey AT, et al. British Association of Spine Surgeons standards of care for cauda equina syndrome. *Spine J* 2015;15(3 Suppl):S2-4. doi: 10.1016/j.spinee.2015.01.006
7. Society of British Neurological Surgeons. Standards of Care for Established and Suspected Cauda Equina Syndrome, 2009, version 4.
8. Society of British Neurological Surgeons. SBNS Care Quality Statement. 2015.
9. Ahn UM, Ahn NU, Buchowski JM, et al. Cauda equina syndrome secondary to lumbar disc herniation: a meta-analysis of surgical outcomes. *Spine* 2000;25(12):1515-22.
10. Kohles SS, Kohles DA, Karp AP, et al. Time-dependent surgical outcomes following cauda equina syndrome diagnosis: comments on a meta-analysis. *Spine* 2004;29(11):1281-7.
11. Korse NS, Jacobs WC, Elzevier HW, et al. Complaints of micturition, defecation and sexual function in cauda equina syndrome due to lumbar disk herniation: a systematic review. *Eur Spine J* 2013;22(5):1019-29. doi: 10.1007/s00586-012-2601-8 .
12. Gardner A, Gardner E, Morley T. Cauda equina syndrome: a review of the current clinical and medico-legal position. *Eur Spine J* 2011;20(5):690-7. doi: 10.1007/s00586-010-1668-3
13. Bell DA, Collie D, Statham PF. Cauda equina syndrome: what is the correlation between clinical assessment and MRI scanning? *Br J Neurosurg* 2007;21(2):201-3. doi: 10.1080/02688690701317144
14. Gleave JR, Macfarlane R. Cauda equina syndrome: what is the relationship between timing of surgery and outcome? *Br J Neurosurg* 2002;16(4):325-8.
15. Srikandarajah N, Boissaud-Cooke MA, Clark S, et al. Does early surgical decompression in cauda equina syndrome improve bladder outcome? *Spine* 2015;40(8):580-3. doi: 10.1097/brs.0000000000000813
16. Crocker M, Fraser G, Boyd E, et al. The value of interhospital transfer and emergency MRI for suspected cauda equina syndrome: a 2-year retrospective study. *Ann R Coll Surg Engl* 2008;90(6):513-6. doi: 10.1308/003588408x301154
17. Hurme M, Alaranta H, Torma T, et al. Operated lumbar disc herniation: epidemiological aspects. *Ann Chir Gynaecol* 1983;72(1):33-6.
18. Podnar S. Epidemiology of cauda equina and conus medullaris lesions. *Muscle Nerve* 2007;35(4):529-31. doi: https://dx.doi.org/10.1002/mus.20696
19. The National Spinal Taskforce. Commissioning Spinal Services - Getting The Service Back On Track, January 2013.

For peer review only - http://bmjopen.bmj.com/site/about/guidelines.xhtml

20. Office for National Statistics. Annual Mid-year Population Estimates, 2010. Statistical Bulletin, 30 June 2011.
21. Brennan PM, Kolias AG, Joannides AJ, et al. The management and outcome for patients with chronic subdural hematoma: a prospective, multicenter, observational cohort study in the United Kingdom. *J Neurosurg* 2016:1-8. doi: 10.3171/2016.8.jns16134
22. Fairbank JC, Pynsent PB. The Oswestry Disability Index. *Spine* 2000;25(22):2940-52
23. Krogh K, Christensen P, Sabroe S, et al. Neurogenic bowel dysfunction score. *Spinal Cord* 2006;44(10):625-31. doi: 10.1038/sj.sc.3101887
24. McGrother CW, Donaldson MM, Shaw C, et al. Storage symptoms of the bladder: prevalence, incidence and need for services in the UK. *BJU Int* 2004;93(6):763-9. doi: 10.1111/j.1464-410X.2003.04721.x
25. McGahuey CA, Gelenberg AJ, Laukes CA, et al. The Arizona Sexual Experience Scale (ASEX): reliability and validity. *J Sex Marital Ther* 2000;26(1):25-40.
26. International Committee of Medical Journal Editors. Recommendations for the Conduct, Reporting, Editing and Publication of Scholarly Work in Medical Journals.

For peer review only

For peer review only - http://bmjopen.bmj.com/site/about/guidelines.xhtml

AUTHORS' CONTRIBUTIONS

JW, IH, SP, and AABJ contributed to the conception of the study, design of the study, writing of the protocol, revision of the protocol, and approving the final version. NS, MP, HR, AKD, PS, NE, PFXS and the BNTRC contributed to the design of the study, revision of the protocol, and approving the final version

FUNDING STATEMENT

This research received no specific grant from any funding agency in the public, commercial or not-for-profit sectors.

COMPETING INTERESTS STATEMENT

JW reports grants from the Wellcome Trust during this study. IH reports grants from the Association of British Neurologists/Patrick Berthoud Charitable Trust during this study. AABJ, SP, NS, MP, HR, AKD, PS, NE, PFXS, and the BNTRC have nothing to disclose.

ACKNOWLEDGEMENTS

We would like to acknowledge the guidance and support in developing this protocol from the study steering committee (Simon Clark, Andreas Demetriades, Gareth Dobson, Niall Eames, Ingrid Hoeritzauer, Aimun Jamjoom, Andraay Leung, Michael Poon, Manjunath Prasad, Savva Pronin, Phil Sell, Nisaharan Srikandarajah, Patrick Statham, Stacey Thomson, Martin Wilby, Julie Woodfield), the British Association of Spinal Surgeons (Mike Hutton, David Cumming, Jake Timothy, Andraay Leung, Chrishan Thakar, Tim Germon), the British Orthopaedic Trainees Assocation (Rory Morrison), the British Neurosurgical Trainee Research Collaborative (Ellie Edlman, Ruichong Ma, Michael Poon), and the sponsor NHS Lothian (Kenneth Scott). We would like to thank all patients at the Department of Clinical Neurosciences in Edinburgh who provided input into the design of the study and the questionnaires.

FIGURE LEGENDS

Figure 1: Study Flow Diagram. Time points for patient reported and clinician reported data collection in UCES.

For peer review only - http://bmjopen.bmj.com/site/about/guidelines.xhtml

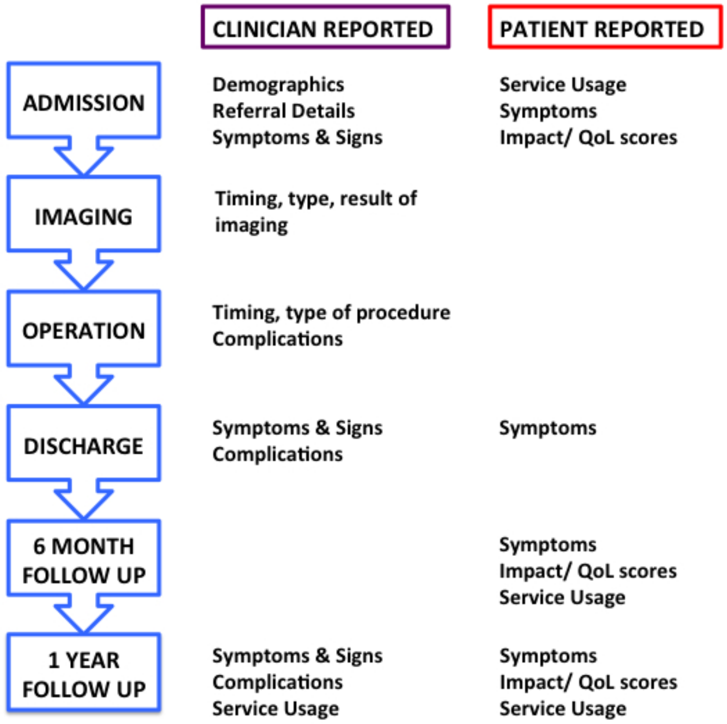

Figure 1: Study Flow Diagram. Time points for patient reported and clinician reported data collection in UCES.

SPIRIT

STANDARD PROTOCOL ITEMS: RECOMMENDATIONS FOR INTERVENTIONAL TRIALS

SPIRIT 2013 Checklist: Recommended items to address in a clinical trial protocol and related documents*

Section/item	Item No	Description	Addressed on page number
**Administrative information**			
Title	1	Descriptive title identifying the study design, population, interventions, and, if applicable, trial acronym	1______________
Trial registration	2a	Trial identifier and registry name. If not yet registered, name of intended registry	2______________
	2b	All items from the World Health Organization Trial Registration Data Set	1, 6,7, 8, 9, 12, 16
Protocol version	3	Date and version identifier	www.bntrc.org.uk/protocols (Most recent date and version number includes current participating sites)
Funding	4	Sources and types of financial, material, and other support	16_____________
Roles and responsibilities	5a	Names, affiliations, and roles of protocol contributors	1,16___________
	5b	Name and contact information for the trial sponsor	12______________
	5c	Role of study sponsor and funders, if any, in study design; collection, management, analysis, and interpretation of data; writing of the report; and the decision to submit the report for publication, including whether they will have ultimate authority over any of these activities	16____________

For peer review only

	5d	Composition, roles, and responsibilities of the coordinating centre, steering committee, endpoint adjudication committee, data management team, and other individuals or groups overseeing the trial, if applicable (see Item 21a for data monitoring committee)	16____________
**Introduction**			
Background and rationale	6a	Description of research question and justification for undertaking the trial, including summary of relevant studies (published and unpublished) examining benefits and harms for each intervention	4,5_________
	6b	Explanation for choice of comparators	N/A_________
Objectives	7	Specific objectives or hypotheses	5___________
Trial design	8	Description of trial design including type of trial (eg, parallel group, crossover, factorial, single group), allocation ratio, and framework (eg, superiority, equivalence, noninferiority, exploratory)	5,6__________
**Methods: Participants, interventions, and outcomes**			
Study setting	9	Description of study settings (eg, community clinic, academic hospital) and list of countries where data will be collected. Reference to where list of study sites can be obtained	5,6 www.bntrc.org.uk/protocols for list of current study sites
Eligibility criteria	10	Inclusion and exclusion criteria for participants. If applicable, eligibility criteria for study centres and individuals who will perform the interventions (eg, surgeons, psychotherapists)	6,7__________
Interventions	11a	Interventions for each group with sufficient detail to allow replication, including how and when they will be administered	7,8,9_________
	11b	Criteria for discontinuing or modifying allocated interventions for a given trial participant (eg, drug dose change in response to harms, participant request, or improving/worsening disease)	N/A__________
	11c	Strategies to improve adherence to intervention protocols, and any procedures for monitoring adherence (eg, drug tablet return, laboratory tests)	N/A__________

For peer review only - http://bmjopen.bmj.com/site/about/guidelines.xhtml

	11d	Relevant concomitant care and interventions that are permitted or prohibited during the trial	N/A_________
Outcomes	12	Primary, secondary, and other outcomes, including the specific measurement variable (eg, systolic blood pressure), analysis metric (eg, change from baseline, final value, time to event), method of aggregation (eg, median, proportion), and time point for each outcome. Explanation of the clinical relevance of chosen efficacy and harm outcomes is strongly recommended	8,9_________
Participant timeline	13	Time schedule of enrolment, interventions (including any run-ins and washouts), assessments, and visits for participants. A schematic diagram is highly recommended (see Figure)	7,8,9, Figure1
Sample size	14	Estimated number of participants needed to achieve study objectives and how it was determined, including clinical and statistical assumptions supporting any sample size calculations	8___________
Recruitment	15	Strategies for achieving adequate participant enrolment to reach target sample size	7___________
**Methods: Assignment of interventions (for controlled trials)**			
Allocation:			
Sequence generation	16a	Method of generating the allocation sequence (eg, computer-generated random numbers), and list of any factors for stratification. To reduce predictability of a random sequence, details of any planned restriction (eg, blocking) should be provided in a separate document that is unavailable to those who enrol participants or assign interventions	N/A_________
Allocation concealment mechanism	16b	Mechanism of implementing the allocation sequence (eg, central telephone; sequentially numbered, opaque, sealed envelopes), describing any steps to conceal the sequence until interventions are assigned	N/A__________
Implementation	16c	Who will generate the allocation sequence, who will enrol participants, and who will assign participants to interventions	N/A__________
Blinding (masking)	17a	Who will be blinded after assignment to interventions (eg, trial participants, care providers, outcome assessors, data analysts), and how	N/A__________
	17b	If blinded, circumstances under which unblinding is permissible, and procedure for revealing a participant's allocated intervention during the trial	N/A__________

For peer review only - http://bmjopen.bmj.com/site/about/guidelines.xhtml

Methods: Data collection, management, and analysis

Data collection methods	18a	Plans for assessment and collection of outcome, baseline, and other trial data, including any related processes to promote data quality (eg, duplicate measurements, training of assessors) and a description of study instruments (eg, questionnaires, laboratory tests) along with their reliability and validity, if known. Reference to where data collection forms can be found, if not in the protocol	7,8__________
	18b	Plans to promote participant retention and complete follow-up, including list of any outcome data to be collected for participants who discontinue or deviate from intervention protocols	7___________
Data management	19	Plans for data entry, coding, security, and storage, including any related processes to promote data quality (eg, double data entry; range checks for data values). Reference to where details of data management procedures can be found, if not in the protocol	8,9,10,11,12
Statistical methods	20a	Statistical methods for analysing primary and secondary outcomes. Reference to where other details of the statistical analysis plan can be found, if not in the protocol	8,9__________
	20b	Methods for any additional analyses (eg, subgroup and adjusted analyses)	8,9__________
	20c	Definition of analysis population relating to protocol non-adherence (eg, as randomised analysis), and any statistical methods to handle missing data (eg, multiple imputation)	8,9__________

Methods: Monitoring

Data monitoring	21a	Composition of data monitoring committee (DMC); summary of its role and reporting structure; statement of whether it is independent from the sponsor and competing interests; and reference to where further details about its charter can be found, if not in the protocol. Alternatively, an explanation of why a DMC is not needed	N/A__________
	21b	Description of any interim analyses and stopping guidelines, including who will have access to these interim results and make the final decision to terminate the trial	N/A_________
Harms	22	Plans for collecting, assessing, reporting, and managing solicited and spontaneously reported adverse events and other unintended effects of trial interventions or trial conduct	N/A__________
Auditing	23	Frequency and procedures for auditing trial conduct, if any, and whether the process will be independent from investigators and the sponsor	N/A___________

For peer review only - http://bmjopen.bmj.com/site/about/guidelines.xhtml

Ethics and dissemination

Research ethics approval	24	Plans for seeking research ethics committee/institutional review board (REC/IRB) approval	12____________
Protocol amendments	25	Plans for communicating important protocol modifications (eg, changes to eligibility criteria, outcomes, analyses) to relevant parties (eg, investigators, REC/IRBs, trial participants, trial registries, journals, regulators)	13__________
Consent or assent	26a	Who will obtain informed consent or assent from potential trial participants or authorised surrogates, and how (see Item 32)	9,10__________
	26b	Additional consent provisions for collection and use of participant data and biological specimens in ancillary studies, if applicable	N/A__________
Confidentiality	27	How personal information about potential and enrolled participants will be collected, shared, and maintained in order to protect confidentiality before, during, and after the trial	11,12_________
Declaration of interests	28	Financial and other competing interests for principal investigators for the overall trial and each study site	16___________
Access to data	29	Statement of who will have access to the final trial dataset, and disclosure of contractual agreements that limit such access for investigators	13___________
Ancillary and post-trial care	30	Provisions, if any, for ancillary and post-trial care, and for compensation to those who suffer harm from trial participation	12__________
Dissemination policy	31a	Plans for investigators and sponsor to communicate trial results to participants, healthcare professionals, the public, and other relevant groups (eg, via publication, reporting in results databases, or other data sharing arrangements), including any publication restrictions	13__________
	31b	Authorship eligibility guidelines and any intended use of professional writers	13__________
	31c	Plans, if any, for granting public access to the full protocol, participant-level dataset, and statistical code	13___________

Appendices

Informed consent materials	32	Model consent form and other related documentation given to participants and authorised surrogates	www.bntrc.org.uk/ protocols__
Biological specimens	33	Plans for collection, laboratory evaluation, and storage of biological specimens for genetic or molecular analysis in the current trial and for future use in ancillary studies, if applicable	N/A__________

*It is strongly recommended that this checklist be read in conjunction with the SPIRIT 2013 Explanation & Elaboration for important clarification on the items. Amendments to the protocol should be tracked and dated. The SPIRIT checklist is copyrighted by the SPIRIT Group under the Creative Commons "Attribution-NonCommercial-NoDerivs 3.0 Unported" license.

For peer review only
